# Liver fibrosis-induced muscle atrophy is mediated by elevated levels of circulating TNFα

**DOI:** 10.1038/s41419-020-03353-5

**Published:** 2021-01-07

**Authors:** Tamaki Kurosawa, Momo Goto, Noriyuki Kaji, Satoshi Aikiyo, Taiki Mihara, Madoka Ikemoto-Uezumi, Masashi Toyoda, Nobuo Kanazawa, Tatsu Nakazawa, Masatoshi Hori, Akiyoshi Uezumi

**Affiliations:** 1 Laboratory of Veterinary Pharmacology, Department of Veterinary Medical Sciences, Graduate School of Agriculture and Life Sciences, Tokyo University, 1-1-1 Yayoi, Bunkyo-ku, Tokyo, 113-8657 Japan; 2 Muscle Aging and Regenerative Medicine, Tokyo Metropolitan Institute of Gerontology (TMIG), 35-2 Sakae-cho, Itabashi-ku, Tokyo, 173-0015 Japan; 3 Laboratory of Veterinary Pharmacology, School of Veterinary Medicine, Azabu University, 1-17-71, Fuchinobe, Chuo-ku, Sagamihara, Kanagawa 252-5201 Japan; 4 Vascular Medicine, TMIG, Tokyo, Japan; 5 Department of Surgery, Tokyo Metropolitan Geriatric Hospital and Institute of Gerontology (TMGHIG), 35-2 Sakae-cho, Itabashi-ku, Tokyo, 173-0015 Japan; 6 Seibo Hospital, 2-5-1 Nakaochiai Shinjyuku, Tokyo, 161-8521 Japan

**Keywords:** Mechanisms of disease, Diseases

## Abstract

Liver cirrhosis is a critical health problem associated with several complications, including skeletal muscle atrophy, which adversely affects the clinical outcome of patients independent of their liver functions. However, the precise mechanism underlying liver cirrhosis-induced muscle atrophy has not been elucidated. Here we show that serum factor induced by liver fibrosis leads to skeletal muscle atrophy. Using bile duct ligation (BDL) model of liver injury, we induced liver fibrosis in mice and observed subsequent muscle atrophy and weakness. We developed culture system of human primary myotubes that enables an evaluation of the effects of soluble factors on muscle atrophy and found that serum from BDL mice contains atrophy-inducing factors. This atrophy-inducing effect of BDL mouse serum was mitigated upon inhibition of TNFα signalling but not inhibition of myostatin/activin signalling. The BDL mice exhibited significantly up-regulated serum levels of TNFα when compared with the control mice. Furthermore, the mRNA expression levels of *Tnf* were markedly up-regulated in the fibrotic liver but not in the skeletal muscles of BDL mice. The gene expression analysis of isolated nuclei revealed that *Tnf* is exclusively expressed in the non-fibrogenic diploid cell population of the fibrotic liver. These findings reveal the mechanism through which circulating TNFα produced in the damaged liver mediates skeletal muscle atrophy. Additionally, this study demonstrated the importance of inter-organ communication that underlies the pathogenesis of liver cirrhosis.

## Introduction

The liver is involved in various physiological processes, including nutrient metabolism, immune response, detoxification, and digestion. The physiological healing responses to liver injury involve the activation of inflammatory responses and production of extracellular matrix proteins. However, chronic liver injury transforms these physiological healing responses into morbid processes, which lead to the disruption of tissue architecture, loss of hepatic function, and consequently liver cirrhosis^1–3^. Globally, there is increased incidence of mortality due to liver cirrhosis or associated complications among adults^4,5^. The major complications associated with liver fibrosis include hepatocellular carcinoma, variceal bleeding, ascites, and encephalopathy, which contribute to short survival and poor quality of life (QOL)^6^. In addition to these conditions, muscle atrophy has attracted growing attention recently as a serious complication caused by liver fibrosis^7^. Liver cirrhosis-induced muscle atrophy decreases the QOL of patients and is associated with poor prognosis^8–10^. The Japan Society of Hepatology has established the clinical assessment criteria for muscle atrophy associated with liver disease^11^.

Skeletal muscles mainly comprise large cylindrical cells called myofibers, which are the largest cells in the human body. There are more than 600 skeletal muscles in the human body and each muscle tissue is made up of hundreds of myofibers. Skeletal muscles account for approximately 30% of the whole-body weight in healthy adults. The primary function of skeletal muscle is to mediate body movement through contraction, which enables physical activity and exercise. Additionally, skeletal muscles are a major reservoir of amino acids in the human body and thus play a central role in whole-body protein metabolism^12^. Skeletal muscles are crucial for maintaining the QOL. The progressive decline in skeletal muscle mass and function with ageing results in a condition termed sarcopenia. Sarcopenia not only reduces exercise capacity but also deteriorates general health, and therefore is one of the major determinants of healthy life expectancy. Several studies have indicated that muscle atrophy increases the mortality among the elderly^13–16^. Sarcopenia can be classified as primary and secondary sarcopenia^17^. Ageing is the solo aetiological factor for primary sarcopenia, whereas there are several aetiological factors for secondary sarcopenia in addition to ageing. Liver disease-associated sarcopenia is categorised into secondary sarcopenia and the link between liver disease and sarcopenia has become an increasingly important clinical issue^7,18^. However, the mechanism that connect these two morbid conditions is not fully understood.

The signalling pathway involved in skeletal muscle atrophy has been well studied. Several extracellular molecules, such as tumour necrosis factor alpha (TNFα), TNF-like weak inducer of apoptosis (TWEAK), glucocorticoid and myostatin (MSTN) are reported to induce muscle atrophy^19,20^. Patients with liver fibrosis exhibit up-regulated expression of MSTN and TNFα^21–23^. MSTN, which is a member of the transforming growth factor beta superfamily, is a negative regulator of muscle mass. Mice with deletion of the *Mstn* gene exhibit a marked increase in the overall muscle mass^24^, which indicates the negative regulatory effect of MSTN on skeletal muscles. TNFα, which is a member of the TNF superfamily, is involved in diverse biological processes, such as inflammation, apoptosis, and tumorigenesis. TNFα exerts its biological function through activation of various intracellular signalling pathways, including JNK, Erk1/2, p38 MAPK, and NF-κB signalling pathways. The activated NF-κB signalling pathway is reported to induce muscle atrophy through upregulation of MuRF1, a key E3 ubiquitin ligase that mediates sarcomeric protein degradation^25^. Despite these studies, the factors that induce liver fibrosis-induced muscle atrophy have not been completely understood.

In this study, the mechanism underlying liver fibrosis-induced muscle atrophy was elucidated using the bile duct ligation (BDL)-induced liver fibrosis mouse model. The findings of this study indicated that the atrophy-inducing factor is in the serum of BDL mice. The inhibition assays revealed that serum TNFα is an atrophy-inducing factor. Additionally, this study demonstrated that TNFα is produced in the fibrotic liver but not in the atrophied muscle of BDL mice. These findings demonstrate that circulating TNFα is critical for inter-organ communication, which is important for the pathogenesis of liver cirrhosis-induced muscle atrophy.

## Materials and methods

### Mice

C57BL/6 and *Pdgfra*^*EGFP*^ mice were purchased from Japan SLC (Shizuoka, Japan) and Jackson Laboratory (stock# 007669, Bar Harbor, ME, USA), respectively. The mice were supplied with food and water ad libitum and maintained under constant temperature and humidity in a 12-h dark/light cycle. All animal experiments were approved by the Experimental Animal Care and Use Committee of University of Tokyo and Tokyo Metropolitan Institute of Gerontology and were performed in accordance with the ARRIVE guidelines.

### BDL model

Eight-week-old male C57BL/6 mice and *Pdgfra*^*EGFP*^ mice were randomly divided into BDL and sham operation groups. The BDL group mice were subjected to biliary obstructions as previously described^26^. The common bile duct was exposed through an abdominal incision and ligated at two points under anaesthesia. The sham operation mice underwent the same surgical procedure but the bile duct was not ligated. The abdomen was closed, and the anaesthesia antagonist was used to awaken the mice. The anaesthesia antagonist dose was adjusted according to the method described in a previous study^27^. At day 7 or 21 post-operation, the hindlimb muscles and liver were excised and snap-frozen in liquid nitrogen-chilled isopentane.

### Etanercept treatment in vivo

Etanercept (Mochida Pharmaceutical Co., Ltd, Tokyo, Japan) was reconstituted in PBS to a final concentration of 1 mg/ml. Mice were intraperitoneally injected with etanercept (10 mg/kg) 24 h before surgery, immediately after surgery, and at days 2, 4, and 6 post-operation. Control mice were injected with the same volume of PBS.

### Grip strength

Grip strength of the BDL and sham operation group mouse forelimbs was measured using a grip strength measuring device (GPM-101V, MELQUEST, Toyama, Japan) and (MK380-M, Muromachi Kikai, Tokyo, Japan). The measurement was repeated five times for each mouse and the maximum grip strength was recorded.

### Histology

For immunofluorescence staining, the freshly frozen liver tissues were sectioned into 8-μm-thick sections using cryostat (Leica Microsystems, Wetzlar, Germany). The sections were fixed with 4% paraformaldehyde (PFA) for 5 min and blocked with Protein Block Serum-Free (Agilent Technologies, Santa Clara, CA, USA) for 5 min. Next, the sections were incubated with the rabbit anti-collagen type I antibody (1:50; Abcam, Cambridge, UK, Cat# ab21286) at 4 °C overnight, followed by incubation with Alexa Fluor 594-conjugated donkey anti-rabbit IgG (1:1000; Jackson ImmunoResearch, West Grove, PA, USA) antibody for 1 h. The sections were counterstained with 4′,6-diamidino-2-phenylindole (DAPI) (1:5000; DOJINDO, Kumamoto, Japan) and mounted with SlowFade Diamond antifade reagent (Thermo Fisher Scientific, Waltham, MA, USA). The fluorescent images were captured using a fluorescence microscope DM6000FS (Leica Microsystems). The confocal images were captured using the confocal laser scanning microscopy system TCS SP8 (Leica Microsystems). Collagen type I-positive areas were quantitated using WinROOF2015 (Mitani Corporation, Tokyo, Japan). To analyse the muscle fibre cross-sectional area (CSA), the 8-μm-thick frozen sections were cut at a position 2.5 mm from the proximal end of tibialis anterior (TA) muscle. The sections were fixed in ice-cooled acetone for 5 min and incubated with the rat anti-laminin α2 antibody (clone 4H8-2; 1:200; Santa Cruz Biotechnology, Dallas, TX, USA, Cat# sc-59854) at 4 °C overnight, followed by incubation with Alexa Fluor 488-conjugated donkey anti-rat IgG (1:1000; Jackson ImmunoResearch) antibody. Images of entire sections were captured using a fluorescence microscope BZ-X710 (Keyence, Osaka, Japan). CSA and the number of myofibers were quantified using the Hybrid Cell Count software (Keyence). For sequential staining, the cover glasses were removed after immunofluorescence staining, and the same sections were stained with haematoxylin and eosin (H&E). The images of the sections were captured using a microscope (Optiphot-2, Nikon, Tokyo, Japan) equipped with a CCD camera (DXM1200C, Nikon).

### Blood chemistry tests

The blood samples collected from the BDL or sham groups were incubated at room temperature for 2 h. To collect the serum, the blood samples were centrifuged at 2000*g* for 20 min. The serum level of TNFα was determined using the Mouse TNFα Quantikine HS ELISA kit (R&D systems, Minneapolis, MN, USA). The serum levels of non-esterified fatty acids (NEFA) were determined by an enzymatic colorimetric method (LabAssay^TM^ NEFA, Wako, Tokyo, Japan).

### Human myotube culture and atrophy assay

The experiments on primary human cells were approved by the Ethical Committee at Tokyo Metropolitan Geriatric Hospital and Institute of Gerontology. All subjects provided their written informed consent to participate in the study. Small fragments of muscle tissue were obtained from two human subjects. The human myoblasts were isolated from the gluteus medius or gastrocnemius as previously described^28^. The myoblasts were seeded on Matrigel-coated (CORNING, Corning, NY, USA) 96-well imaging plates (Cell Carrier-96 ultra, PerkinElmer, Waltham, MA, USA) and cultured in growth medium (GM; Dulbecco’s modified Eagle’s medium (DMEM) supplemented with 20% foetal bovine serum (FBS), 1% penicillin–streptomycin, and 2.5 ng/mL basic fibroblast growth factor (Hygieia Bioscience, Osaka Japan)). The cells were cultured till 90% confluency at 37 °C in 5% CO_2_ and 3% O_2_. The effects of serum from BDL or sham operation group mice and inhibitors on mature myotubes were examined following a previously described method^29^ with minor modifications. The differentiation of myoblasts into myotubes was induced using the differentiation medium (DM; DMEM supplemented with 5% horse serum and 1% penicillin–streptomycin). The cells were cultured at 37 °C, 5% CO_2_, and 20% O_2_ for 5 days. The DM was replaced with GM and the cells were cultured for 48 h. The cells were then serum-starved for 4 h before treatment with sera or inhibitors. The myotubes were incubated with the serum from BDL or sham operation group in the presence or absence of follistatin (R&D Systems) or TNFRII-Fc protein (R&D Systems) for 3 days. The myotubes were fixed with 4% PFA for 5 min and permeabilised with 0.1% Triton X-100. Next, the myotubes were blocked and incubated with mouse anti-myosin heavy chain (MyHC) antibody (clone MF20; 1:2; DSHB) at 4 °C overnight, followed by incubation with the Alexa Fluor 647-conjugated donkey anti-mouse IgG (1:1000; Jackson ImmunoResearch) antibody for 1 h. The images of the myotubes were captured using the High Content Imaging system IN Cell Analyzer 6000 (Cytiva, Amersham, UK). The MyHC-positive areas were analysed using the IN Cell investigator v.1.9.2 software (Cytiva).

### Analysis of the effect of mouse TNFα on human myotubes

Human myoblasts were induced to differentiate into myotubes in Matrigel-coaled 96-well plates as described above. After serum starvation for 4 h, myotubes were incubated with or without recombinant mouse TNFα (R&D systems, Minneapolis, MN, USA). The myotubes were fixed with 4% PFA for 5 min, treated with 0.1% Triton X-100, blocked and incubated with mouse anti-myosin heavy chain (MyHC) antibody (clone MF20; 1,2; DSHB) at 4 °C overnight. After incubation with the Alexa Fluor 488-conjugated donkey anti-mouse IgG (1:1000; Jackson ImmunoResearch) antibody for 2 h, the images of the myotubes were captured using the High Content Imaging system IN Cell Analyzer 6000 (Cytiva, Amersham, UK). The MyHC-positive areas were analysed using the IN Cell investigator v.1.9.2 software (Cytiva).

### Capillary-based immunoassay

Cells were lysed in 1× lysis buffer (RayBiotech, Peachtree Corners, GA, USA) supplemented with cOmplete ULTRA (Roche, Basel, Schweiz) and PhosSTOP (Roche) tablets on ice. The lysates were rotated 15 min and sonicated for 5 min at 4 °C. After 15 min centrifugation at 14,000 r.p.m. at 4 °C, the supernatants were collected as protein lysate samples. The protein concentration of each lysate was determined using Pierce 660 nm Protein Assay (Thermo Fisher Scientific, Waltham, MA, USA) according to the manufacturer’s protocol. The protein concentration was adjusted to 0.5 or 1 mg/mL using 0.1× sample buffer 2 (ProteinSimple, San Jose, CA) for the detection of MyHC or MyoD and myogenin, respectively. Protein separation and detection were performed using an automated capillary electrophoresis system Wes (ProteinSimple) with capillary cartridges for 12–230 kDa (ProteinSimple). Following antibodies were used: anti-MyHC (clone MF20; 1:20; DSHB), anti-MyoD (clone EPR6653-131; 1:50; Abcam, Cambridge, UK) and anti-myogenin (clone EPR4789; 1:50; Abcam). Signals were analysed and visualised using Compass software (ProteinSimple).

### Nucleus isolation from liver

Fresh frozen livers of *Pdgfra*^*EGFP*^ mice subjected to BDL or sham operation were sectioned into 100-μm-thick sections using cryostat on day 21 post-operation. The sections (20 mg) were collected into homogenisation tubes (BMS, Tokyo, Japan) containing 3 mm zirconia beads (BMS). The samples were homogenised in 1 mL of hypotonic buffer comprising 250 mM sucrose, 10 mM KCl, 5 mM MgCl_2_, 10 mM Tris-HCl (pH 8.0), 25 mM HEPES, 0.1 mM dithiothreitol, 0.1% Triton X-100, 0.2 U/μL RNase inhibitor (Takara, Shiga, Japan), and protease inhibitor cocktail (Roche, Basel, Schweiz) for 10 s using a Shakeman homogeniser (BMS). The samples were incubated on ice for 15 min and homogenised again for 10 s using the homogeniser. The lysate was filtered through 100- and 40-μm cell strainers (CORNING), followed by filtration through a 5-mL filter cap FACS tube (CORNING). DAPI (1:5000; DOJINDO) was used to stain the DNA. From each population, 10,000 nuclei were collected using FACSAriaII (BD Biosciences, San Jose, CA, USA). Total RNA was extracted from the samples using the RNeasy Micro kit (Qiagen, Hilden, Germany). The cDNA was synthesised from the extracted RNA using Maxima H reverse transcriptase (Thermo Fisher Scientific).

### RNA extraction and real-time PCR analysis

Freshly frozen TA muscles and livers were sliced to a thickness of 10 μm using a cryostat, and the 100 sections were collected to extract RNA. Total RNA was extracted using the RNeasy Mini kit (Qiagen). The extracted RNA was reverse transcribed into cDNA using the QuantiTect Reverse Transcription Kit (Qiagen). Real-time PCR analysis was performed using the AriaMx Real-time PCR system (Agilent Technologies) or Takara Thermal Cycler Dice Real-Time System (Takara) with THUNDERBIRD SYBR qPCR Mix (QPS-201, TOYOBO, Osaka, Japan) or SYBR Premix Ex Taq II (Takara). The PCR conditions were as follows: 95 °C for 60 s, followed by 40 cycles of 95 °C for 15 s, 60 °C for 20 s, and 72 °C for 30 s for tissue samples, or 94 °C for 30 s, followed by 50 cycles of 94 °C for 5 s, 60 °C for 20 s, and 72 °C for 12 s for isolated nucleus samples. Dissociation curve analysis was performed after amplification. The relative expression level of the target genes was calculated using the ΔΔCt method. The *Cmas* or *Gapdh* genes were used as reference genes to analyse the target gene expression levels in the tissues or isolated nucleus, respectively. The following primers were used for real-time PCR analysis:

For mouse

*Acta2* forward 5′-CGTGCCTATCTATGAGGGCTATG-3′ and *Acta2* reverse 5′-ACAATCTCACGCTCGGCAGTA-3′;

*Adgre1* forward 5′-GTGGAAGCATCCGAGACACA-3′ and *Adgre1* reverse 5′-GCTATGGCCAAGGCAAGACA-3′;

*Ccn2* forward 5′-CCACCCGAGTTACCAATGAC-3′ and *Ccn2* reverse 5′-ACAGGCTTGGCGATTTTAGG-3′;

*Cmas* forward 5′-CAAAGGCATCCCACTGAAGA-3′ and *Cmas* reverse 5′-CCCACACACTCTGGAAGACC-3′;

*Col1a1* forward 5′-CCGAACCCCAAGGAAAAGAA-3′ and *Col1a1* reverse 5′-GTGGACATTAGGCGCAGGAA-3′;

*Col1a2* forward 5′-GCTGGTGTAATGGGTCCTCC-3′ and *Col1a2* reverse 5′-CGACCGGCATCTCCATTAGG-3′;

*Fbxo32* forward 5′-ACTTCTCGACTGCCATCCTG-3′ and *Fbxo32* reverse 5′-GATGTGAGCTGTGACTTTGCTATC-3′;

*Fn1* forward 5′-CCCAGCTCACTGACCTAAGC-3′ and *Fn1* reverse 5′-TTCTCCTGCCGCAACTACTG-3′;

*Gapdh* forward 5′-CCTGGAGAAACCTGCCAAGTATG-3′ and *Gapdh* reverse 5′-AGAGTGGGAGTTGCTGTTGAAGTC-3′;

*Hnf4a* forward 5′-AGAGGTTCTGTCCCAGCAGATC-3′ and *Hnf4a* reverse 5′-CGTCTGTGATGTTGGCAATC-3′;

*Kdr* forward 5′-GACGTCGACATAGCCTCCAC-3′ and *Kdr* reverse 5′-TCGGTGATGTACACGATGCC-3′;

*Lrat* forward 5′-ATGGCTCTCGGATCAGTCCA-3′ and *Lrat* reverse 5′-GCCAGGCCTGTGTAGACAAT-3′;

*Pdgfra* forward 5′-TCAGCTGTCTCCTCACAGGG-3′ and *Pdgfra* reverse 5′-ACTCTCCCCAACGCATCTCA-3′;

*Tgfb1* forward 5′-TGAGTGGCTGTCTTTTGACG-3′ and *Tgfb1* reverse 5′-CGTGGAGTTTGTTATCTTTGCTG-3′;

*Tnf* forward 5′-CCACCACGCTCTTCTGTCTAC-3′ and *Tnf* reverse 5′-GAGGGTCTGGGCCATAGAACT-3′;

*Tnfrs1a*: forward 5′-GAAGTTGTGCCTACCTCCTCC-3′ and reverse 5′-GCAAGATAACCAGGGGCAAC-3′

*Tnfrs1b*: forward 5′-CCAGCCAAACTCCAAGCATC-3′ and reverse 5′-ATGTCACTCCAACAATCAGACC-3′

*Mstn* forward 5′-TGCTGTAACCTTCCCAGGAC-3′ and *Mstn* reverse 5′-GATTCCGTGGAGTGCTCATC-3′;

*Trim63* forward 5′-TGGAAACGCTATGGAGAACC-3′ and *Trim63* reverse 5′-AAGATGTCGTTGGCACACTTC-3′.

For human

*GAPDH*: forward 5′-ACCCACTCCTCCACCTTTGA-3′ and reverse 5′- TTGCTGTAGCCAAATTCGTTG -3′

*TNFRSF1A*: forward 5′-CCCTTCAGAAGTGGGAGGACAG-3′ and reverse 5′-CGAATTCCTTCCAGCGCAACG-3′

*TNFRSF1B*: forward 5′-TTGGACTGATTGTGGGTGTG-3′ and reverse 5′-TTATCGGCAGGCAAGTGAGG-3′

*MSTN*: forward 5′-GGAGAAGATGGGCTGAATCCG-3′ and reverse 5′-CAGCATCGTGATTCTGTTGAGTG-3′.

### Statistics

The effect size for each study was estimated based on a previous study^23^ or preliminary studies. The sample sizes were determined using sample size calculations from the IACUC at Boston university for the type I error rate = 0.05 and power = 0.8. No data show significant deviation from normal distribution and no data were excluded in this study. Muscle weight measurements were performed in a blinded manner and other experiments were not blinded. All statistical analyses were performed using Prism (version 8.4.1; GraphPad Software, San Diego, CA, USA). Equality of variances between the groups was analysed by *F*-test or Bartlett’s test. The differences between two groups were analysed using the two-sided unpaired Student’s *t*-test and Welch’s correction was applied when variances between the groups was not equal. The differences between more than two groups were analysed using one-way analysis of variance (ANOVA), followed by Dunnett’s post hoc test, or Kruskal–Wallis non-parametric test followed by Dunn’s post hoc test when variances between the groups was not equal. The differences were considered statistically significant when the *P* value was less than 0.05.

## Results

### BDL-induced liver fibrosis

**Fig. 1:**
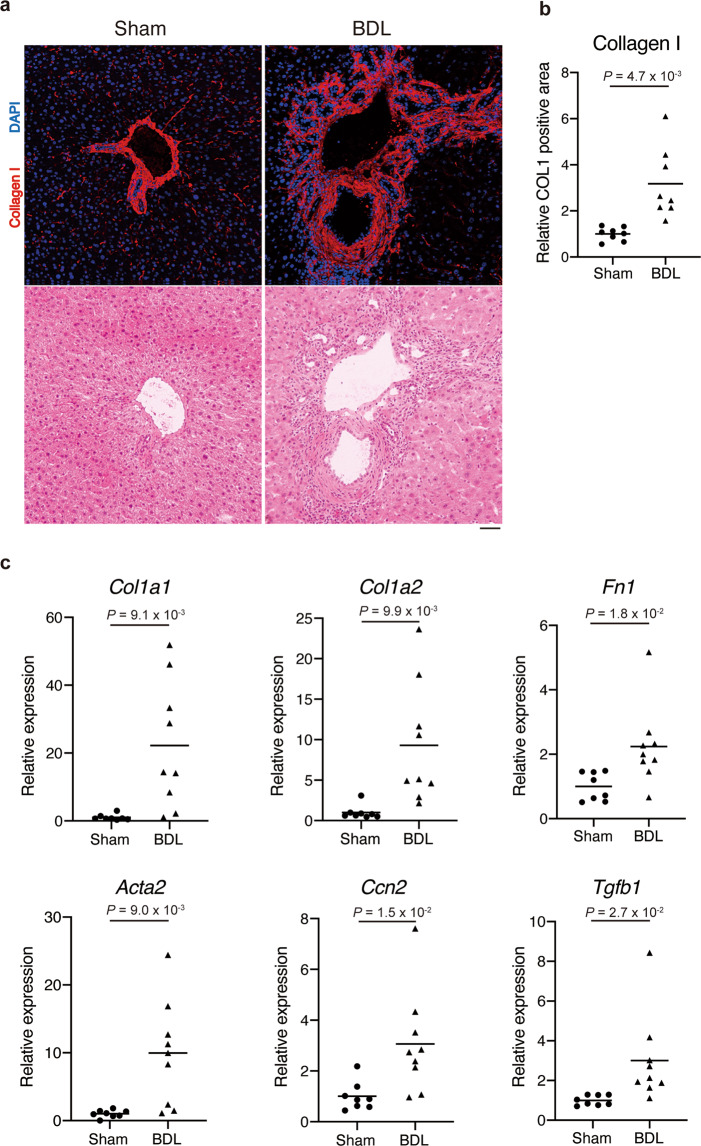
BDL induces liver fibrosis in mice. The common bile duct of eight-week-old male mice was ligated to induce liver fibrosis. **a** The sections derived from livers excised on day 7 post-operation were subjected to immunofluorescence staining for collagen I and DAPI (upper) and subsequently to H&E staining (lower). **b** The collagen I-positive area was quantified. *n* = 8 mice for each group. **c** The hepatic expression levels of fibrosis-related genes (*Col1a1, Col1a2, Fn1, Acta2, Ccn2*, and *Tgfb1*) were quantified on day 7 post-operation. *n* = 8 mice for sham and *n* = 9 mice for BDL. Data represent individual data points and the means. Data were analysed using the two-sided unpaired t-test and Welch’s correction. Scale bar, 50 μm (**a**).

BDL is the most common method used to induce liver fibrosis^30,31^. Eight-week-old male mice were subjected to BDL surgery to induce liver fibrosis. At day 7 post-operation, the connective tissue around the bile ducts thickened and the regular lobular structures in the liver were disrupted (Fig. 1a, lower panels). The accumulation of collagen I around the duct structures in BDL group mice was higher than that in sham operation group mice (Fig. 1a, upper panels and b). The hepatic expression levels of fibrosis-related genes were significantly up-regulated in BDL group mice (Fig. 1c). At day 21 post-operation, the collagen I-positive fibrotic region was spread throughout the liver in BDL mice (Supplementary Fig. 1). This indicated that BDL efficiently induces liver fibrosis in mice and that the fibrotic changes in the liver are already manifested at least at day 7 post-operation.

### BDL induces skeletal muscle atrophy and weakness

**Fig. 2:**
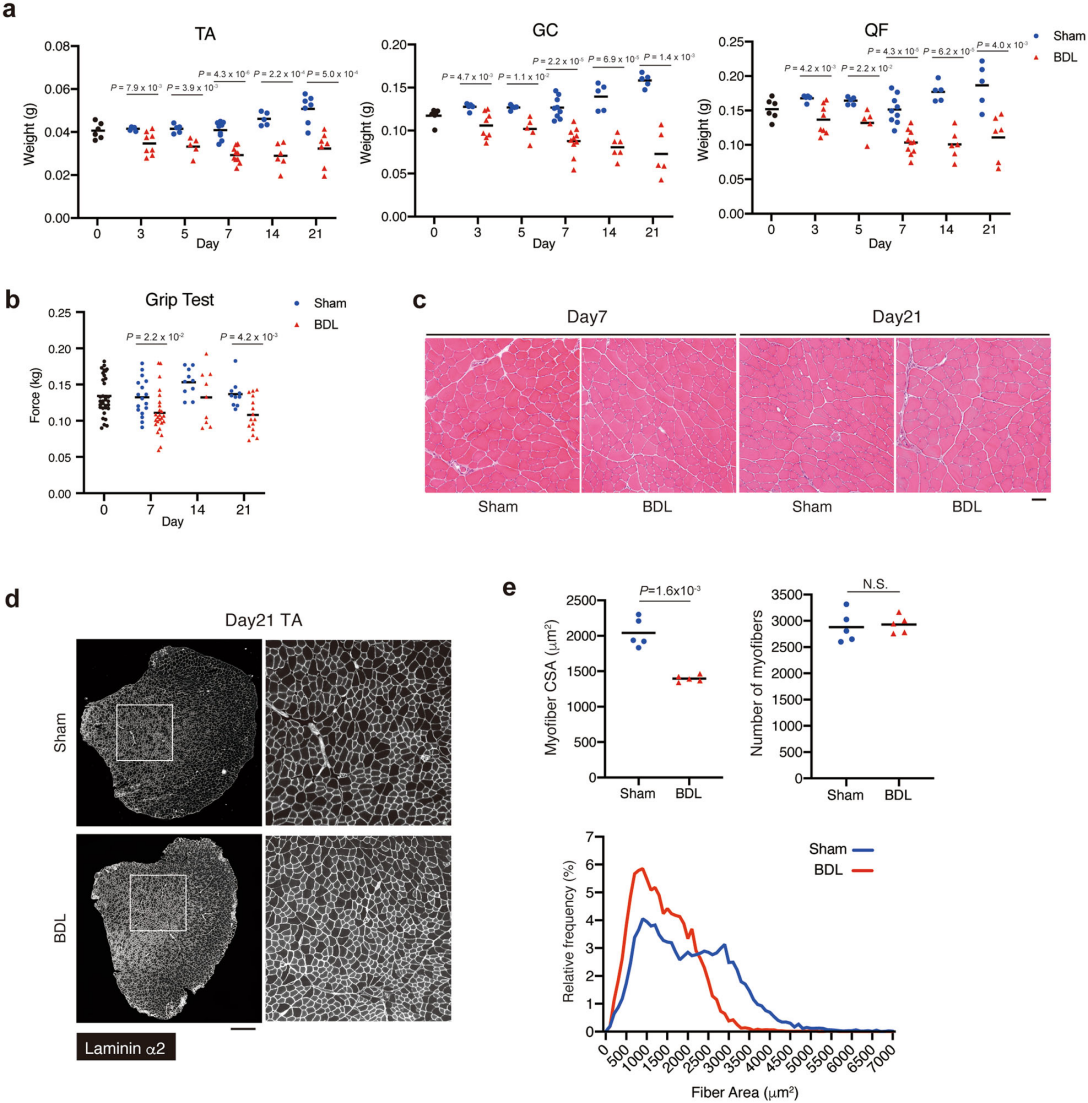
BDL induces skeletal muscle atrophy and weakness. **a** The muscle weight of the BDL or sham group was measured at the time points indicated. TA tibialis anterior, GC gastrocnemius, QF quadriceps femoris. For TA, *n* = 6 at day 0 post-operation and *n* = 5, 5, 9, 5, and 7 (sham) and *n* = 8, 5, 10, 6, and 7 (BDL) at days 3, 5, 7, 14, and 21 post-operation, respectively. For GC, *n* = 6 at day 0 post-operation and *n* = 5, 5, 9, 5, and 5 (sham) and *n* = 8, 5, 10, 6, and 5 (BDL) at days 3, 5, 7, 14, and 21 post-operation, respectively. For QF, *n* = 6 at day 0 post-operation and *n* = 5, 5, 9, 5, and 5 (sham) and *n* = 8, 5, 10, 6, and 7 (BDL) at days 3, 5, 7, 14, and 21 post-operation, respectively. **b** Grip strength of BDL or sham group mice was measured at the indicated time points. *n* = 33 mice at day 0 post-operation. *n* = 17, 10, and 10 (sham) and *n* = 23, 9, and 15 (BDL) mice at days 7, 14, and 21 post-operation, respectively. **c** H&E staining of TA cross-sections from each group at days 7 and 21 post-operation. **d** Images of TA cross sections at day 21 post-operation stained for laminin α2 are shown. Right panels show magnified views of the boxed regions on left panels. **e** Myofiber cross-sectional area (CSA) and the number of myofibers in TA muscle were quantified (upper). Myofiber CSA distribution in TA muscle is shown as a histogram (lower). *n* = 5 mice. Data represent individual data points and the means. Data were analysed using the two-sided unpaired *t*-test. Scale bars, 50 μm (**c**) and 500 μm (**d**).

The phenotype of skeletal muscles during the progression of liver fibrosis induced by BDL was examined. The weight of muscles, including the TA, gastrocnemius (GC), and quadriceps femoris (QF) muscles, decreased in BDL mice at day 3 post-operation. The difference in muscle weight between the BDL and control mice tended to increase after day 3 post-operation (Fig. 2a). The muscle strength was evaluated using the grip test. The change in muscle weight was consistent with the change in grip strength in BDL mice (Fig. 2b). During the process of muscle atrophy, neither the infiltration of inflammatory cells nor myofiber necrosis were observed in both BDL and control mice even at day 21 post-operation (Fig. 2c). Next, the CSA of muscle fibres and number of myofibers were measured. Although each myofiber exhibited atrophy, the number of myofibers was unaffected (Fig. 2d, e). These results indicate that BDL induces liver fibrosis, which promotes muscle atrophy and weakness.

### Serum from BDL mice induces myotube atrophy

**Fig. 3:**
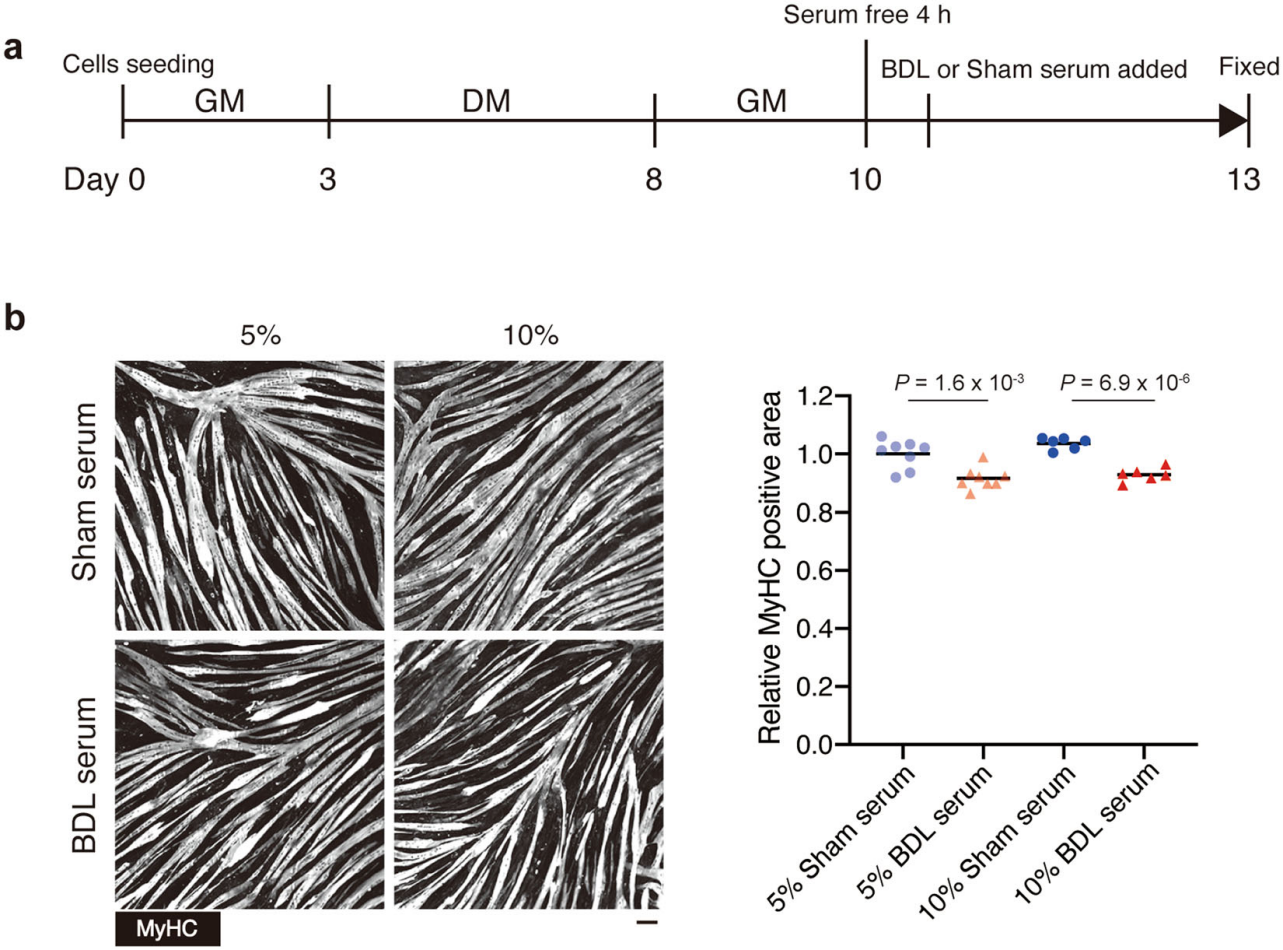
Serum from BDL mice induces myotube atrophy. **a** Experimental design for examining the effect of serum factors on mature human myotubes. **b** Serum-treated myotubes were stained for MyHC (left) and the MyHC-positive area was quantified (right). *n* = 8 or 6 independent wells for 5% or 10% serum treatment, respectively. Data represent individual data points and the means. Data were analysed using the two-sided unpaired *t*-test. Scale bar, 100 μm (**b**).

As muscle atrophy appeared to be a secondary pathological change following liver fibrosis, we hypothesised that atrophy-inducing factors produced in the fibrotic liver are delivered to the skeletal muscle via the bloodstream. To verify this hypothesis, the cultured myotubes were incubated with the serum collected from BDL or control mice. The serum samples were collected on day 7 post-operation as muscle loss and muscle atrophy were clearly evident at this time point. We first tested C2C12 mouse myogenic cell line, but myotubes derived from C2C12 cells tended to peel off after treatment with mouse serum for unknown reasons (Supplementary Fig. 2). In contrast, human primary myotubes were stable in culture and therefore suitable for the evaluation of myotube atrophy in vitro. The well-differentiated myotubes derived from highly purified primary human myoblasts were incubated with the serum from BDL or control mice (Fig. 3a). At day 3 post-serum treatment, myosin heavy chain (MyCH) staining revealed that myotubes cultured with BDL mouse serum were significantly thinner than those cultured with control mouse serum (Fig. 3b). Expression levels of MyHC, MyoD1, and myogenin were also examined by capillary-based immunoassay but there was no significant difference in the expression levels of these proteins between sham serum- and BDL serum-treated cells (Supplementary Fig. 3), suggesting that morphological analysis based on MyHC staining is more suitable than protein level analysis to assess myotube atrophy. Muscle atrophy induced by 10% BDL mouse serum was severer than that induced by 5% serum (Fig. 3b, *P* = 1.6 × 10^−3^ in 5% culture vs *P* = 6.9 × 10^−6^ in 10% culture). These data indicate that the muscle atrophy-inducing factors are present in the serum of mice with liver fibrosis.

### TNFα signalling inhibition alleviates myotube atrophy induced by serum from BDL mice

**Fig. 4:**
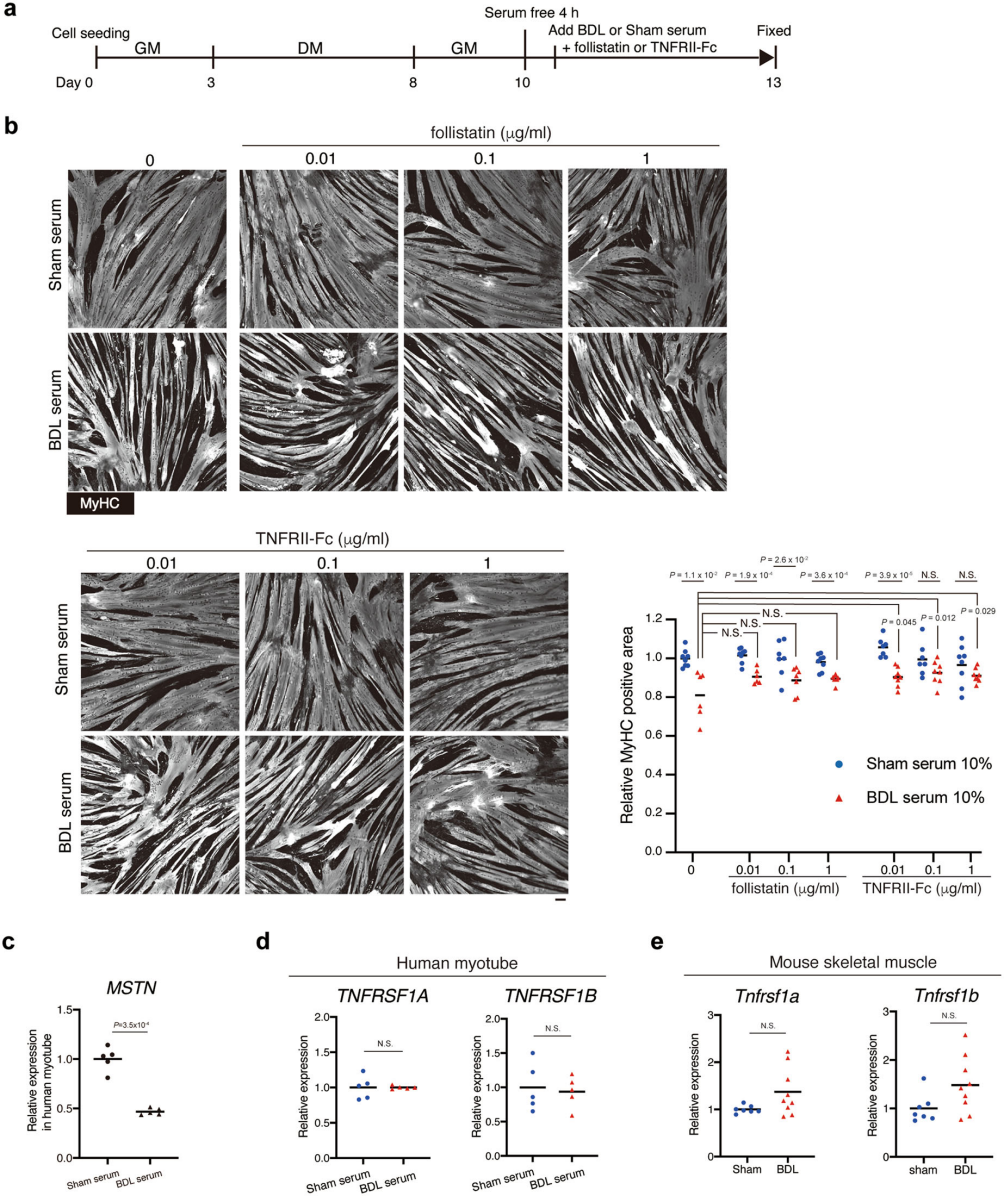
TNFα is a causative substance of BDL mouse serum-induced myotube atrophy. **a** Experimental design for examining the effects of MSTN or TNFα inhibition on mature human myotubes treated with BDL serum. **b** BDL or sham mouse serum-treated human myotubes were treated with follistatin or TNFRII-Fc at the concentrations indicated. Myotubes were stained for MyHC and the MyHC-positive area was quantified. **c** The expression levels of *MSTN* in the human myotubes treated with sham or BDL serum were quantified. **d** The expression levels of TNF receptor genes (*TNFRSF1A* and *TNFRSF1B*) in the human myotubes treated with sham or BDL serum were quantified. **e** The expression levels of TNF receptor genes (*Tnfrsf1a* and *Tnfrsf1b*) in TA muscles from sham or BDL operation mice were quantified. *n* = 8 (sham) and *n* = 6 (BDL) independent wells for control (no addition of follistatin or TNFRII-Fc), *n* = 7, 7, and 8 (sham) and *n* = 6, 7, and 7 (BDL) for 0.01, 0.1, and 1 μg/mL of follistatin, respectively. *n* = 7, 7, and 8 (sham) and *n* = 8, 8, and 8 (BDL) for 0.01, 0.1, and 1 μg/mL of TNFRII-Fc, respectively (**b**), *n* = 5 independent wells (**c**, **d**), *n* = 7 for sham and 9 for BDL mice (**e**). Data represent individual data points and the means. Data were analysed using the two-sided unpaired *t*-test, *t*-test with Welch’s correction, or ANOVA, followed by Dunnett’s post hoc test. NS not significant. Scale bar, 100 μm (**b**).

We sought to identify atrophy-inducing factors that is present in serum derived from the BDL mice. BDL induces loss of adipose tissue (Supplementary Fig. 4a), which may in turn lead to increase in serum levels of free fatty acids. Although fatty acids were reported to induce apoptosis or atrophy in muscle cells^32,33^, it was also reported that lipid overload does not induce lipotoxicity in skeletal muscle^34^. To clarify the involvement of fatty acids in muscle atrophy induced by BDL, serum levels of non-esterified fatty acids (NEFA) were measured. Consistent with previous report^35^, NEFA levels did not differ significantly between control and BDL mice except for day 3 of BDL (Supplementary Fig. 4b). We next focused on other atrophy-related factors, MSTN and TNFα, because upregulation of these two factors was observed during liver fibrosis^21–23^. The role of MSTN and TNFα signalling pathways in inducing muscle atrophy was examined using follistatin and TNFRII-Fc chimera protein. Follistatin directly binds to MSTN, activin, and GDF11, which inhibits the binding of these ligands to their receptors. TNFRII-Fc efficiently binds to the TNFα ligand and inhibits the TNFα signalling pathway by functioning as a decoy receptor. Using the same experimental design as used in Fig. 3, the myotube cultures were co-incubated with follistatin or TNFRII-Fc and BDL or control mouse serum (Fig. 4a). Treatment with follistatin did not markedly affect myotube atrophy. BDL mouse serum-induced myotube atrophy was observed even at the highest treatment concentration of follistatin (Fig. 4b). Downregulation rather than upregulation of *MSTN* expression was observed upon BDL serum treatment, further suggesting that MSTN signalling is not involved in BDL serum-induced myotube atrophy (Fig. 4c). In contrast, TNFRII-Fc suppressed BDL mouse serum-induced myotube atrophy (Fig. 4b). The stronger suppression of atrophy appeared to be achieved with the higher concentration of TNFRII-Fc protein (Fig. 4b). Although there were relatively large variations in MyHC area of myotubes that had been treated with TNFRII-Fc in addition to sham serum for unknown reasons, myotubes treated with TNFRII-Fc and BDL serum showed a statistically significant increase in MyHC area compared to myotubes treated with BDL serum alone (Fig. 4b, red triangle). Because human myotubes were used to investigate the effect of BDL mouse serum, we confirmed cross-reactivity of human cells to mouse TNFα by treating human myotubes with recombinant mouse TNFα. Addition of mouse TNFα to culture medium clearly induced human myotube atrophy (Supplementary Fig. 5), indicating that human myotubes can react to mouse TNFα. Expression of receptors for TNFα was confirmed in both human myotubes and mouse TA muscle, and expression levels of these receptors did not change after BDL serum treatment or BDL operation (Fig. 4d, e). These results indicate that atrophy-inducing effect of BDL serum can be attributed at least in part to TNFα contained therein.

### TNFα expression is up-regulated in the fibrotic liver and not in the skeletal muscle of BDL mice

**Fig. 5:**
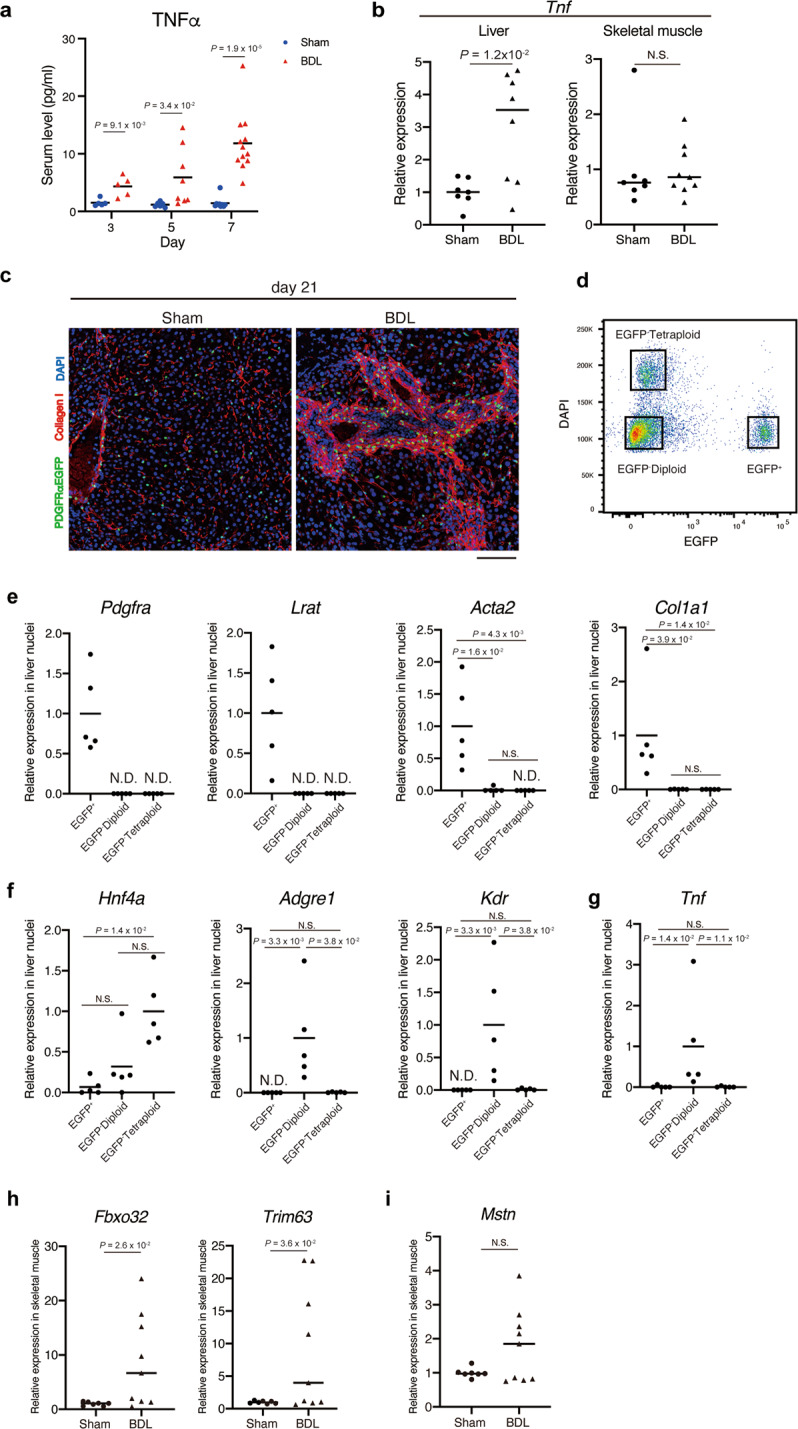
*Tnf* expression is up-regulated in the liver but not in the skeletal muscle of BDL mice. **a** Serum levels of TNFα at day 3, 5, and 7 post-operation were measured using ELISA. **b** The expression level of *Tnf* was quantified in the liver (left) and skeletal muscle (right) at day 7 post-operation. **c** Liver sections of *Pdgfrα*^*EGFP*^ mice at day 21 post-operation were subjected to immunofluorescence staining for collagen I and DAPI. **d** A representative FACS plot for nuclei isolation. Nuclei were isolated from freshly frozen fibrotic livers of *Pdgfrα*^*EGFP*^ mice using FACS. Three gates were set as indicated in the plot to isolate EGFP^+^, EGFP^−^ diploid, and EGFP^−^ tetraploid nuclei. **e** The expression levels of hepatic stellate cell markers (*Pdgfra* and *Lrat*) and fibrosis markers (*Acta2* and *Col1a1*) were quantified. **f** The expression levels of the hepatocyte marker (*Hnf4a*), Kupffer cell marker (*Adgre1*), and endothelial cell marker (*Kdr*) were quantified. **g** Quantitative analysis of *Tnf* gene expression. **h** The expression levels of *Fbxo32* and *Trim63* in the skeletal muscle were quantified. **i** The expression levels of *Mstn* in the skeletal muscle were quantified. *n* = 5, 6, and 9 (sham) and 5, 8, and 12 (BDL) mice for day 3, 5, and 7, respectively (**a**), *n* = 7 (sham) and 8 (BDL) mice for the liver, *n* = 7 (sham) and 9 (BDL) mice for the skeletal muscle (**b**, **h**, **i**), and *n* = 5 mice (**e**–**g**). Data represent individual data points and the means. Data were analysed using the two-sided unpaired *t*-test, *t*-test with Welch’s correction, or Kruskal–Wallis non-parametric test followed by Dunn’s post hoc test. NS not significant, ND not detected. Scale bar, 100 μm (**c**).

The findings so far have suggested that TNFα present in the bloodstream is the causative factor for skeletal muscle atrophy during liver fibrosis. Consistent with these findings, the serum levels of TNFα continued to increase at least until day 7 of BDL (Fig. 5a) with accompanying gradual decrease in muscle weight (Fig. 2a). Inverse relationship between serum TNFα level and muscle mass further supports the importance of TNFα as a causative factor of liver fibrosis-associated muscle wasting. Next, the site of TNFα production was determined. The gene expression level of *Tnf* in the liver was significantly up-regulated, whereas that in the skeletal muscle was unaffected in BDL mice (Fig. 5b). Additionally, the cells exhibiting *Tnf* expression in the fibrotic liver were examined. A recent study demonstrated that the enzymatic method of cell isolation significantly affects transcriptional and histone modification statuses of isolated cells^36^. Thus, the gene expression was analysed using the isolated nuclei. The fibrotic liver was frozen immediately after harvesting and the nuclei were isolated from the frozen liver to eliminate dissociation-induced transcriptional changes. The isolated nuclei exhibited an intact morphology, which indicated that the nucleus isolation method did not affect the nuclear integrity (Supplementary Fig. 6). Liver-resident macrophages called Kupffer cells were reported to produce TNFα during liver injury^37–39^, while other studies demonstrated that hepatic stellate cells (HSCs), which is the origin of fibrogenic cells in the diseased liver^40^, also produce TNFα^41–45^. To clarify the source of TNFα in our experimental setting, we used the *Pdgfra*^*EGFP*^ mice in which the histone *H2B-EGFP* fusion gene is knocked in at the endogenous *Pdgfra* locus^46^. These mice enable the identification of nuclei of HSCs, which specifically exhibit *Pdgfra* expression^47^. Immunofluorescence staining showed that EGFP^+^ cells accumulated in the fibrosis area where collagen I deposition was prominent in the liver at day 21 post-operation (Fig. 5c). The nuclei from the fibrotic liver were fractionated into the following three populations: EGFP^+^ nuclei, EGFP^−^ diploid nuclei, and EGFP^−^ tetraploid nuclei (Fig. 5d). Specific expression of lecithin-retinol acyltransferase (*Lrat*), a marker of HSCs, and *Pdgfra* in EGFP^+^ nuclei ensures accurate labelling of HSCs by the *Pdgfra*^*EGFP*^ reporter (Fig. 5e). As expected, the expression levels of fibrogenic genes were highly up-regulated in EGFP^+^ nuclei (Fig. 5e). The EGFP^−^ fraction comprises diploid and tetraploid nuclei. The expression of genes related to Kupffer cells (*Adgre1*), endothelial cells (*Kdr*) and hepatocytes (*Hnf4a*) were detected in the EGFP^−^ diploid nuclei. However, the EGFP^−^ tetraploid nuclei exhibited expression of only hepatocyte-related genes (Fig. 5f). Interestingly, the *Tnf* gene was expressed exclusively in the EGFP^−^ diploid nuclei (Fig. 5g), which indicated that non-fibrogenic diploid cells are the source of TNFα in the fibrotic liver. TNFα is reported to induce muscle atrophy through the upregulation of atrophy-related E3 ubiquitin ligases, such as atrogin1 and MuRF1 (refs. ^25,48^). Thus, the expression of *Fbxo32* and *Trim63* genes that encode atrogin1 and MuRF1, respectively, was analysed. The expression levels of *Fbxo32* and *Trim63* were significantly up-regulated in the atrophying muscle of BDL mice at day 7 post-operation (Fig. 5h), implying that the elevated serum level of TNFα leads to muscle atrophy through activation of atrogin1 and MuRF1 pathways. Although follistatin-mediated inhibition of MSTN did not affect myotube atrophy, the expression of *Mstn* in the skeletal muscles of BDL and control mice was analysed. The *Mstn* expression levels in the skeletal muscles were not significantly different between BDL and control mice (Fig. 5i). This suggested that MSTN signalling is not involved in muscle atrophy associated with this particular pathological condition.

### In vivo blockade of TNFα attenuates loss of muscle mass and function without affecting liver fibrosis in BDL mice

**Fig. 6:**
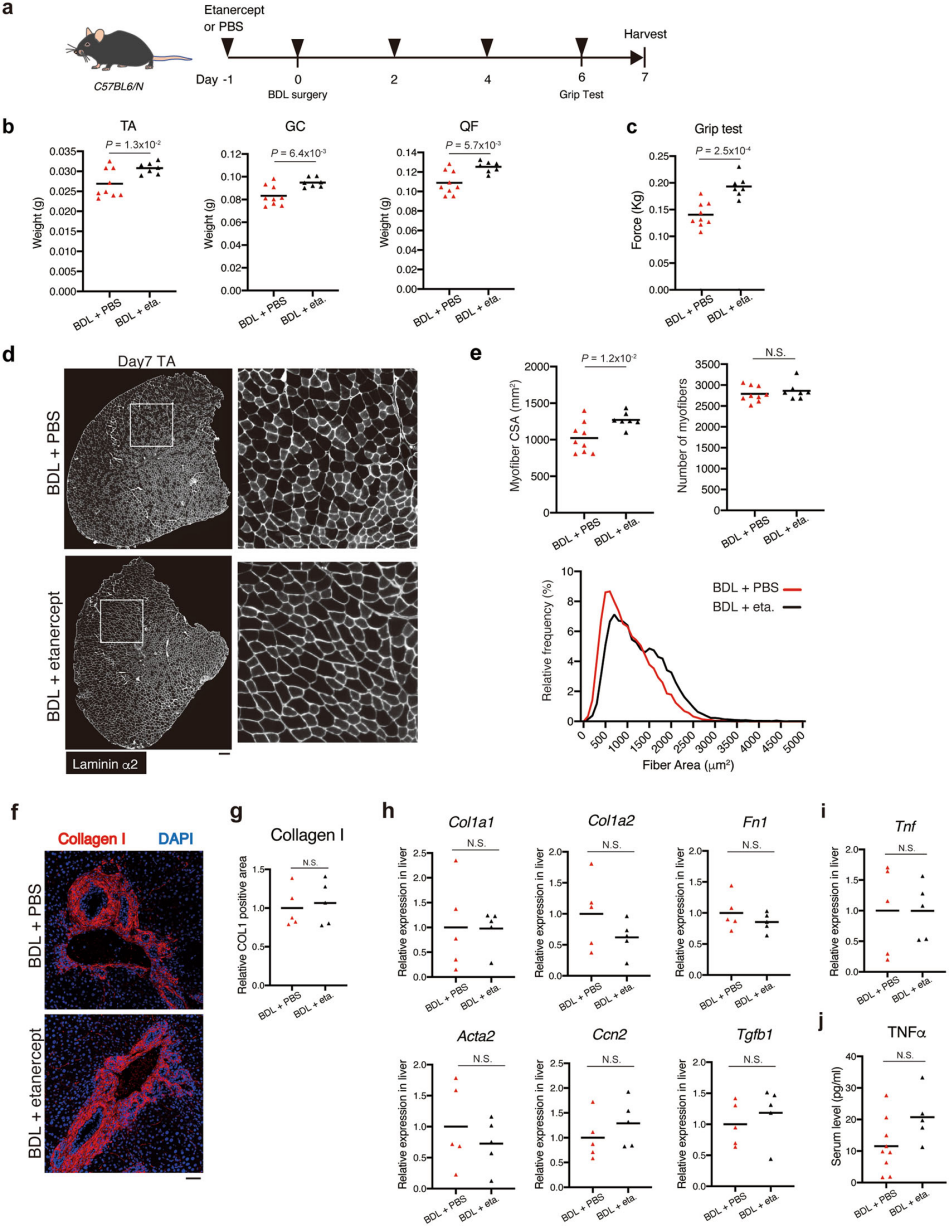
TNFα inhibition alleviates BDL-induced muscle atrophy. **a** Experimental design of TNFα blockade by etanercept. **b**, **c** The muscle weight (**b**) and grip strength (**c**) of the BDL mice treated with PBS or etanercept was measured at day 7. **d** TA cross sections of the BDL mice treated with PBS or etanercept were stained for laminin α2 at day 7 post-operation. Right panels show magnified views of the boxed regions on left panels. **e** Myofiber CSA and the number of myofibers in TA of BDL mice treated with PBS or etanercept were quantified (upper). Myofiber CSA distribution in TA muscle is shown as a histogram (lower). **f** Liver sections of BDL mice treated with PBS or etanercept were subjected to immunofluorescence staining for collagen I and DAPI at day 7 post-operation. **g** The collagen I-positive area was quantified. **h** The hepatic expression levels of fibrosis-related genes (*Col1a1, Col1a2, Fn1, Acta2, Ccn2*, and *Tgfb1*) were quantified on day 7 post-operation of BDL mice treated with PBS or etanercept. **i**, **j** The expression level of *Tnf* in the liver (**i**) and the serum levels of TNFα (**j**) of the BDL mice treated with PBS or etanercept were measured at day 7 post-operation. *n* = 9 mice for PBS group and *n* = 7 for etanercept group (**b**, **c**, **e**). *n* = 5 mice for each group (**g**, **h**, **i**). *n* = 9 mice for PBS group and *n* = 5 for etanercept group (**j**). Data were analysed using the two-sided unpaired *t*-test. Scale bar, 200 μm (**d**) and 50 μm (**f**).

Finally, we performed in vivo blockade of TNFα by etanercept, a clinically approved version of TNFRII-Fc (Fig. 6a). Body weight and serum levels of NEFA were not changed with etanercept treatment (Supplementary Fig. 7). Intriguingly, etanercept treatment attenuated loss of muscle weight and grip strength of BDL-treated mice (Fig. 6b, c). Etanercept treatment led to increase in myofiber CSA without affecting myofiber number of BDL-treated mice (Fig. 6d, e), indicating that myofiber atrophy induced by BDL is alleviated by TNFα inhibition. Next, effect of etanercept on liver fibrosis was examined. Collagen I deposition and expression levels of fibrogenic genes in the liver were not changed with etanercept treatment (Fig. 6f–h). Consistent with the result showing non-inhibitory effect of etanercept on liver fibrosis, the gene expression level of *Tnf* in the liver and the serum levels of TNFα were not affected by etanercept treatment (Fig. 6i, j). Thus, it appears that etanercept does not exert positive effect on skeletal muscle indirectly through therapeutic effect on liver fibrosis, rather it prevents muscle wasting directly by inhibiting TNFα signalling in skeletal muscle where TNFα is acting as an atrophy-inducing factor. Collectively, our results indicate that TNFα signalling is the mediator of in vivo muscle wasting induced by BDL.

## Discussion

**Fig. 7:**
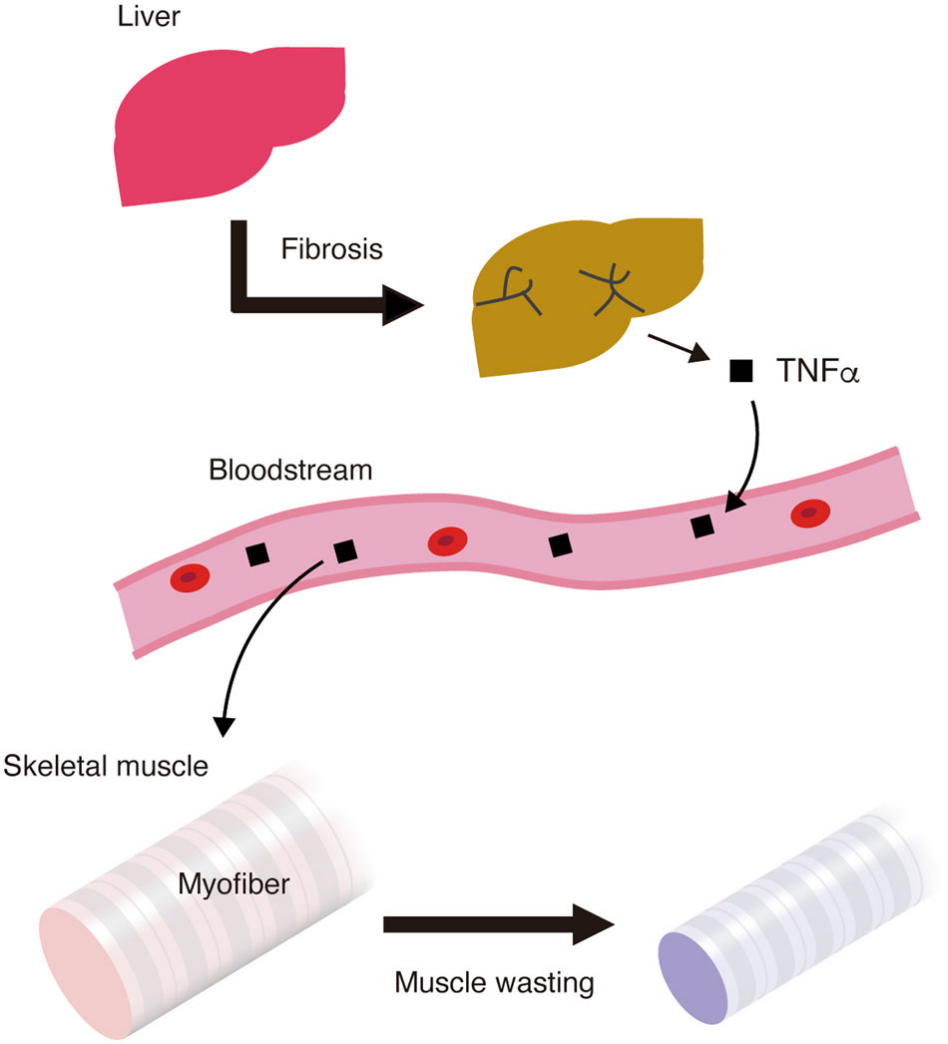
Inter-organ communication mechanism that underlies the pathogenesis of liver fibrosis-induced muscle atrophy. TNFα released from the fibrotic liver, which is delivered to the skeletal muscle through the blood, promotes myofiber atrophy.

This study revealed the mechanism through which TNFα that is produced in the fibrotic liver and transported to the skeletal muscles through the bloodstream induces muscle atrophy (Fig. 7). This inter-organ crosstalk has clinical significance as there is no treatment for liver disease-induced muscle atrophy. The findings of this study demonstrated that TNFRII-Fc protein-mediated inhibition of TNFα signalling protects the human myotubes or mouse skeletal muscle tissues against atrophy induced by the liver fibrosis. Therefore, inhibiting TNFα signalling in the skeletal muscles where TNFα functions as an atrophy-inducing factor can be an effective preventive strategy for liver fibrosis-induced muscle atrophy. Additionally, TNFRII-Fc, also called etanercept, has been clinically approved and used. Thus, TNFRII-Fc can be a potential therapeutic agent for liver disease-associated muscle atrophy. TNFα signalling inhibition has been evaluated as a potential therapeutic strategy for cancer cachexia in clinical trials. However, the results of these clinical trials are inconsistent with one clinical trial reporting beneficial effects, while other clinical trials reporting no improvement^49,50^. Although not tested in liver disease-associated muscle wasting yet, the strategy that depends on TNFα signal inhibition would have an advantage in liver disease over cancer cachexia. The development of liver fibrosis decreased in mice lacking the type I TNF receptor after repeated CCl_4_ injury^51^. A recent study on the intrinsic inactive rhomboid protein 2-mediated regulation of TNFα signalling demonstrated that etanercept alleviates BDL-induced liver fibrosis^52^. Although we could not observe inhibitory effect of etanercept on liver fibrosis in our study, discrepancy between studies described above and ours may be attributed to different time point of the analysis. We examined the effect of TNFα inhibition at day 7 of BDL, while former studies analysed the liver 2 or 4 weeks after the induction of liver fibrosis^51,52^. Therefore, the inhibition of TNFα signalling can exert therapeutic effects on liver fibrosis itself at later stage. Thus, TNFα signalling inhibition may alleviate liver fibrosis and the associated complications by exerting an anti-fibrotic effect on the liver and an anti-atrophic effect on the skeletal muscle.

The nuclei isolated from the fibrotic liver were subjected to gene expression analysis to determine the source of TNFα. Recent studies have revealed that the methods used for cell dissociation affect gene expression. Machado et al.^36^ reported that cell isolation from skeletal muscle through enzymatic digestion markedly affected the transcriptional and histone modification statuses. In the adult kidney, the cell dissociation procedure can damage fragile cells and may not release cells that are embedded within the matrix^53^. Liver is also susceptible to cell dissociation-induced damage. Additionally, it is difficult to recover all cell types with equal integrity and efficiency from the liver tissue^54^. To overcome these limitations, gene expression analysis of isolated intact nuclei is proposed as an alternative approach as nuclei are more resistant to mechanical stress than whole cells and the nuclei can be easily isolated from freshly frozen samples^55^. The analysis of freshly frozen fibrotic liver-derived nuclei revealed that *Tnf* is exclusively expressed in non-fibrotic diploid cell population, which includes *Adgre1*-expressing Kupffer cells and *Kdr*-expressing endothelial cells, as well as diploid hepatocytes. The Kupffer cells are a potential source of TNFα because previous studies have demonstrated the expression of TNFα in Kupffer cells during liver injury^37–39^. Single nucleus RNA sequencing can reveal the TNFα-producing cells in fibrotic liver more precisely although this study did not perform this high-cost experiment.

Giusto et al.^23^ showed upregulation of MSTN in skeletal muscle of BDL mice but the functional significance of MSTN on muscle atrophy was not examined. Additionally, the authors quantified the protein expression level of MSTN relative to that of actin but actin expression was markedly down-regulated in their study^23^, which indicated that the expression of MSTN was overestimated. In our study, we did not see apparent effect of follistatin on myotube atrophy nor statistically significant upregulation of MSTN in BDL muscle. Although MSTN is a best-known negative regulator of muscle mass, early clinical trial in muscular dystrophy that directly targets MSTN by neutralising antibody revealed minimal clinical efficacy^56^. Accordingly, novel strategies have been developed to target the MSTN receptor instead of the ligand^50^. Besides MSTN receptor targeting reagents, follistatin is considered as a powerful alternative because it also inhibits activin and GDF11, which are shown to have negative impact on muscle^29,57,58^. In fact, follistatin-mediated inhibition of MSTN/activin signalling pathway further enhances the muscle mass of MSTN-null mice^59^. Nevertheless, treatment with follistatin did not exert a protective effect against myotube atrophy induced by serum from BDL mice in this study, which indicated that the MSTN/activin signalling pathway is not involved in BDL-induced muscle atrophy.

This study demonstrated that TNFα mediates liver fibrosis-induced muscle atrophy. However, the presence of other mechanisms mediating liver fibrosis-induced muscle atrophy cannot be excluded. One of the limitations of our study is the use of human cells to investigate the effect of mouse serum factor. Although we showed that BDL mouse serum induces human myotube atrophy and human myotubes react to mouse TNFα, our culture system cannot uncover the effect of factors that are contained in BDL mouse serum but do not cross-react to human cells. In addition, TNFα inhibition did not lead to complete rescue from in vitro myotube atrophy and in vivo muscle atrophy. The TNFRII-Fc protein-treated myotubes still showed bit smaller MyHC^+^ area than the control myotubes and etanercept-treated mice could not exhibit the same level of muscle mass as sham operation mice. Therefore, other cytokines not investigated in this study may be involved in liver fibrosis-induced muscle atrophy. In addition to the effects of cytokines, other mechanisms such as hyperammonaemia may also have an adverse impact on skeletal muscle. Ammonia catabolism through ureagenesis is an important metabolic function of the liver. Hepatocellular damage can trigger ureagenesis impairment and consequently lead to hyperammonaemia. Ammonia accumulated in the muscle is reported to activate molecular events that contribute to sarcopenia^60^. Impaired protein synthesis induced by ammonia is considered to be the molecular mechanism underlying hyperammonaemia-induced muscle atrophy^61^. Elucidating the mediators of liver-muscle axis will improve our understanding of the pathogenesis of liver disease-induced muscle atrophy.

In conclusion, this study demonstrated that circulating TNFα, which is released from the fibrotic liver, promotes the atrophy of skeletal muscles during liver fibrosis. Our study shows the molecular mechanism of inter-organ communication that underlies the pathogenesis of liver disease-associated muscle wasting and will provide useful information for designing a therapeutic strategy against this health-threatening complication.

## Supplementary information

Supplementary information
