# Supplementary material for: Liver fibrosis-induced muscle atrophy is mediated by elevated levels of circulating TNFα
Source: Cell Death Dis. 2021 Jan 7;12(1):11. doi: 10.1038/s41419-020-03353-5 (PMC7791043; doi:10.1038/s41419-020-03353-5)
Supplement: Supplementary file 1 — Supplementary information [file 41419_2020_3353_MOESM1_ESM.pdf]

Liver fibrosis-induced muscle atrophy is mediated by elevated levels of circulating TNFα

Tamaki Kurosawa, Momo Goto, Noriyuki Kaji, Satoshi Aikiyo, Taiki Mihara, Madoka Ikemoto-Uezumi, Masashi Toyoda, Nobuo Kanazawa, Tatsu Nakazawa, Masatoshi Hori, and Akiyoshi Uezumi

Summary of supplementary information

Page 1: Cover page

Page 2: Supplementary Fig. 1

Page 3: Supplementary Fig. 2

Page 4: Supplementary Fig. 3

Page 5: Supplementary Fig. 4

Page 6: Supplementary Fig. 5

Page 7: Supplementary Fig. 6

Page 8: Supplementary Fig. 7

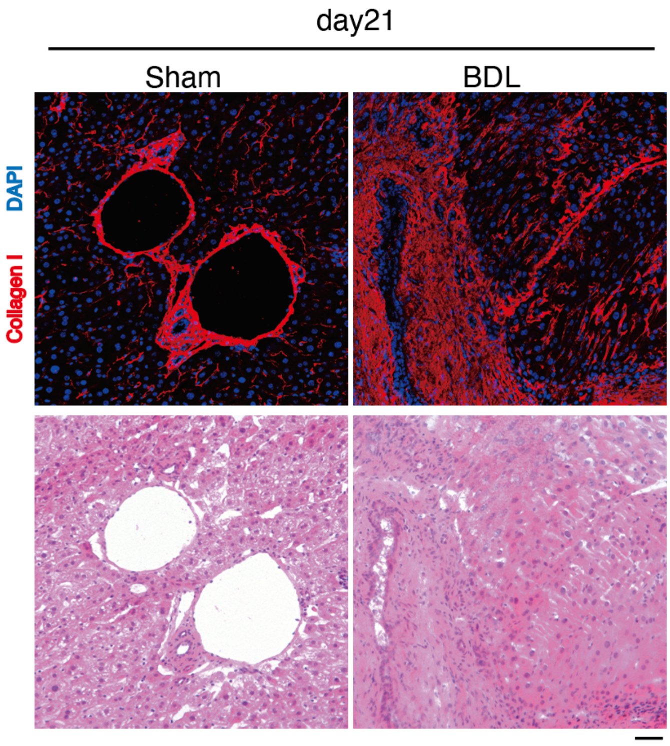

Supplementary Fig. 1

Supplementary Fig. 1: Enhanced liver fibrosis caused by BDL at late time point

The sections of liver excised at day 21 post-operation were subjected to collagen I and DAPI staining (upper), and H&E staining (lower). Scale bar, 50 μm.

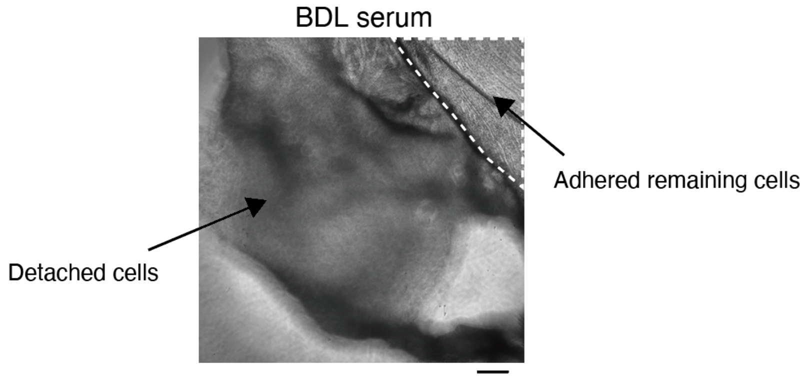

Supplementary Fig. 2

Supplementary Fig. 2: C2C12 myotubes cannot be maintained in mouse serum
C2C12 cells were induced to differentiate into myotubes for 5 days, and then incubated with 10% serum from sham or BDL mice for 2 days. Representative image of BDL serum-treated myotubes was shown. Myotubes were peeled off from the dish and could not be analysed. Scale bar, 250 μm

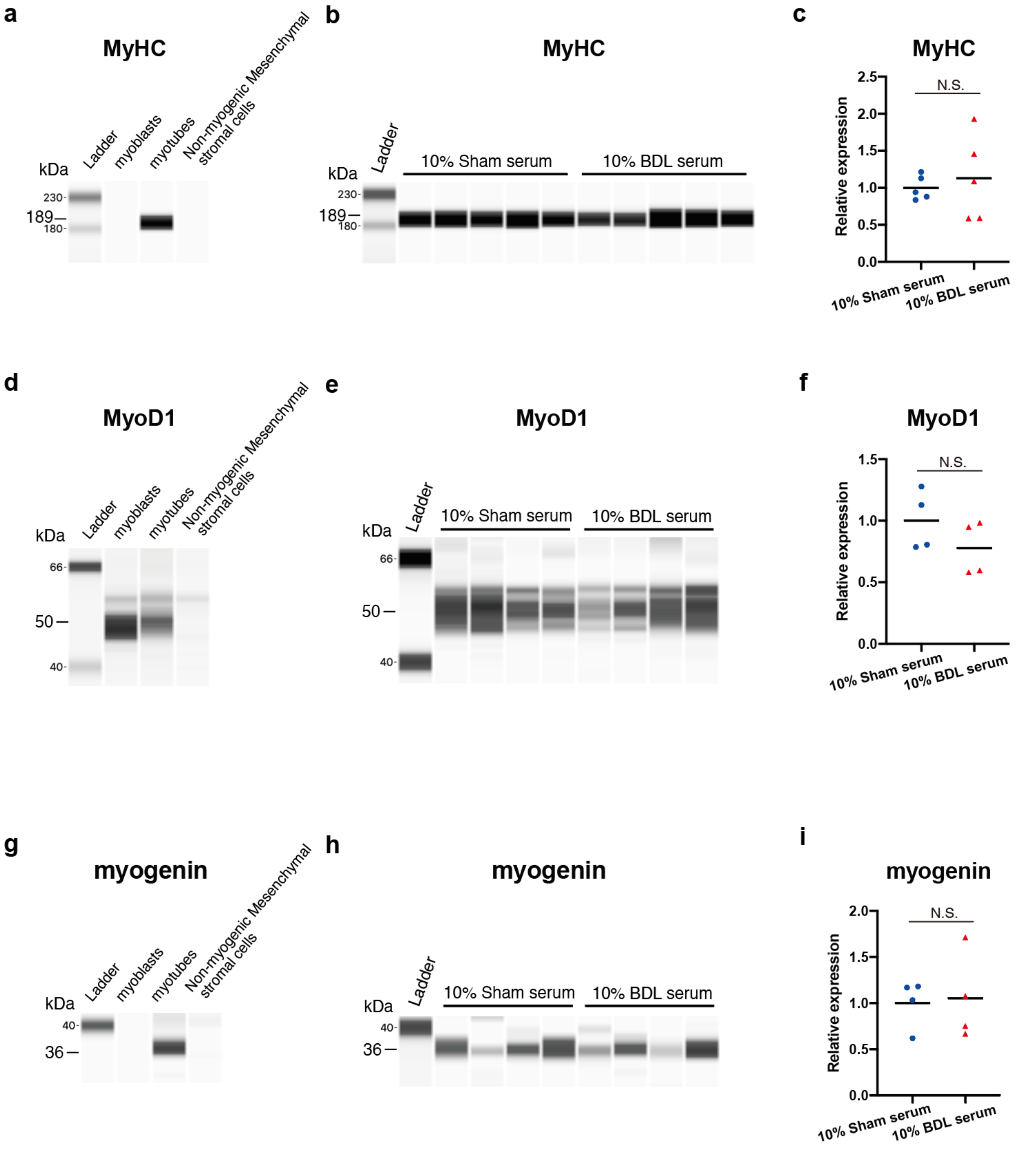

Supplementary Fig. 3

Supplementary Fig. 3: Analysis the protein levels of MyHC and myogenic regulatory factors by capillary-based immunoassay

a, d, g Specificity of antibodies were tested using cell lysates from myoblasts, myotubes, and non-myogenic stromal cells. Note that signals for MyHC and myogenin were detected only in myotube lysate, and MyoD signal was detected in both myoblast and myotube lysates. **b, e, h** Sham serum- or BDL serum-treated myotubes were analysed. **c, f, i** Quantification of MyHC, MyoD, and myogenin levels of **b, e, h**, respectively. No significant differences were observed. n = 5 for MyHC and n = 4 for MyoD and myogenin. Data represent individual data points and the means: two-sided unpaired t-test.

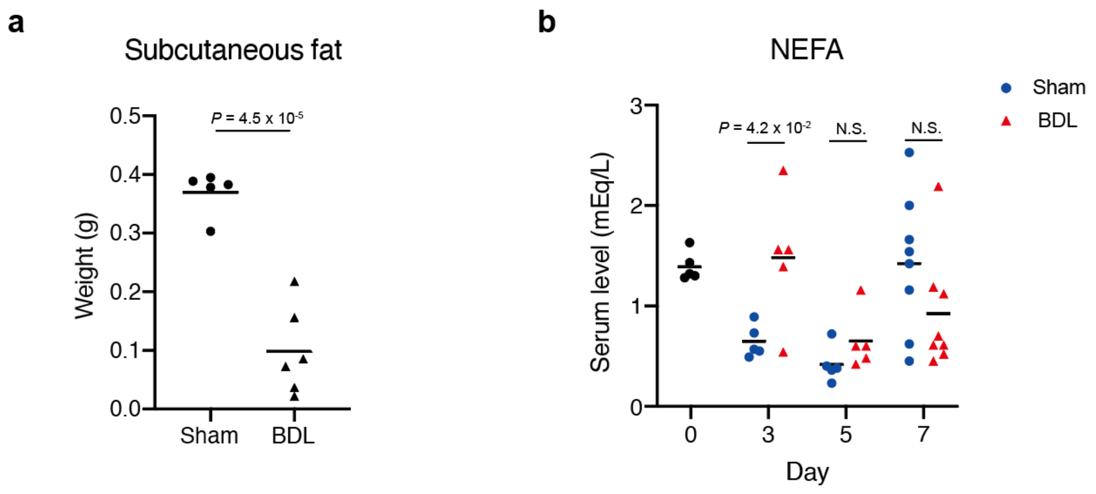

Supplementary Fig. 4

Supplementary Fig. 4: Adipose tissue weight and serum levels of free fatty acids after BDL operation

a Subcutaneous fat weight of sham or BDL operation mice was measured at day 7 post-operation. **b** Serum levels of non-esterified fatty acids (NEFA) were measured at the time points indicated. $n = 5$ (sham) and 6 (BDL) mice (**a**). $n = 5$, 5, 5, and 8 for day 0, 3, 5, and 7, respectively (**b**).

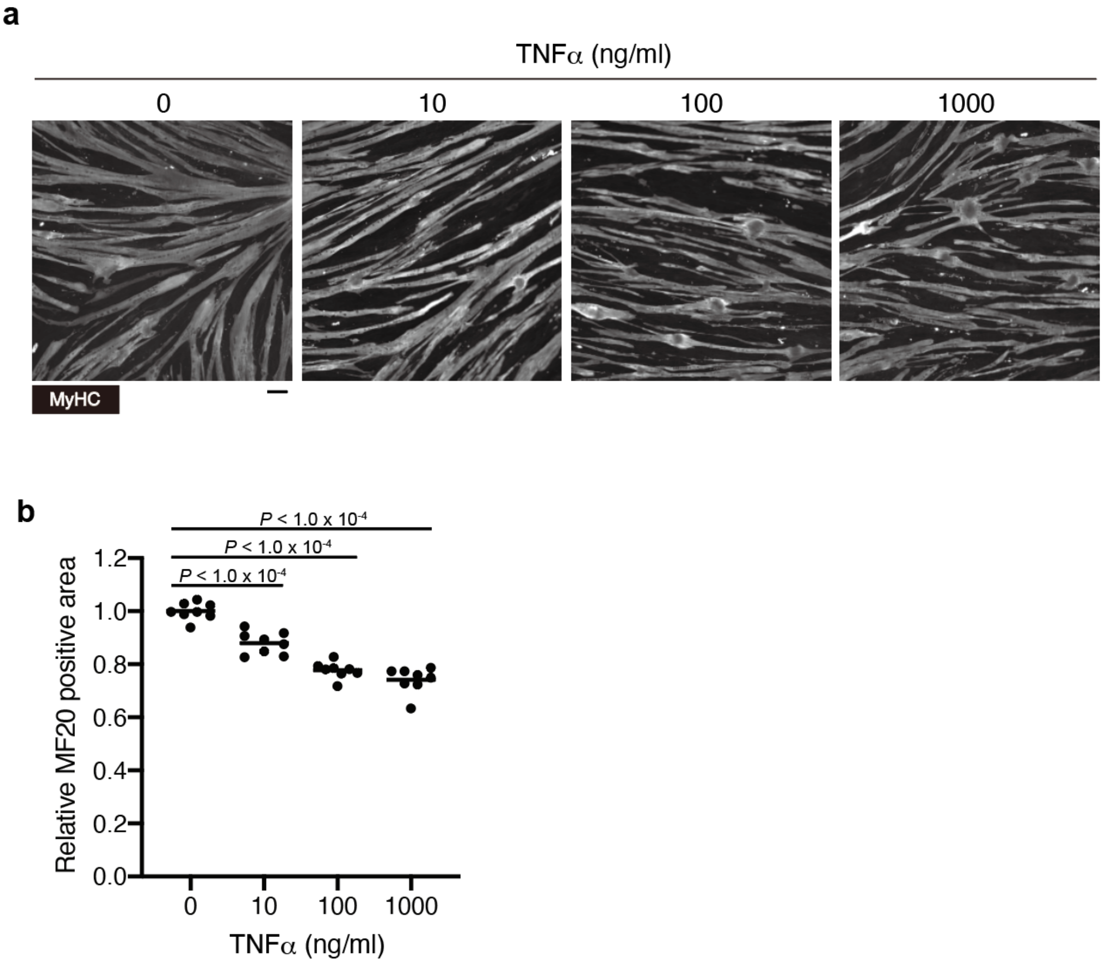

Supplementary Fig. 5

Supplementary Fig. 5: Mouse TNFα induces human myotube atrophy

Well-differentiated human myotubes were treated with recombinant mouse TNFα for 48 hours. Myotubes were stained for MyHC (**a**) and the MyHC-positive area was quantified (**b**). n=8 independent wells. Data represent individual data points and the means. The differences between the groups were analysed using one-way analysis of variance (ANOVA), followed by Dunnett's post-hoc test. Scale bar, 100 μm

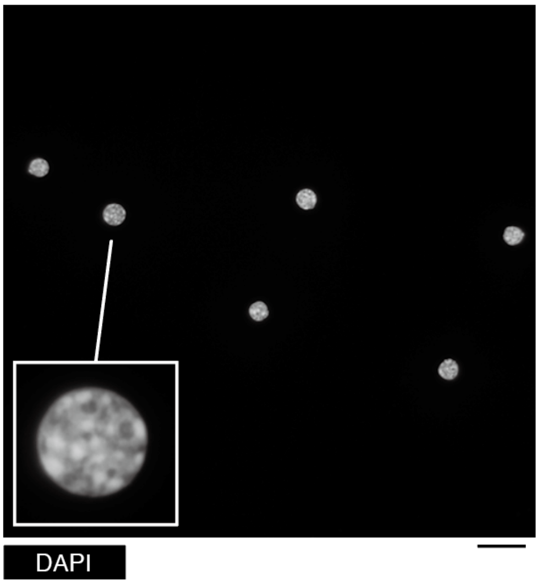

Supplementary Fig. 6

Supplementary Fig. 6: Microscopic analysis of nuclei isolated from freshly frozen liver
Nuclei isolated from freshly frozen liver by FACS were subjected to DAPI staining. Note that heterochromatin where DNA is densely packed and euchromatin where DNA is less condensed are visible, which suggested that the isolated nuclei are structurally intact. Scale bar, 50 µm.

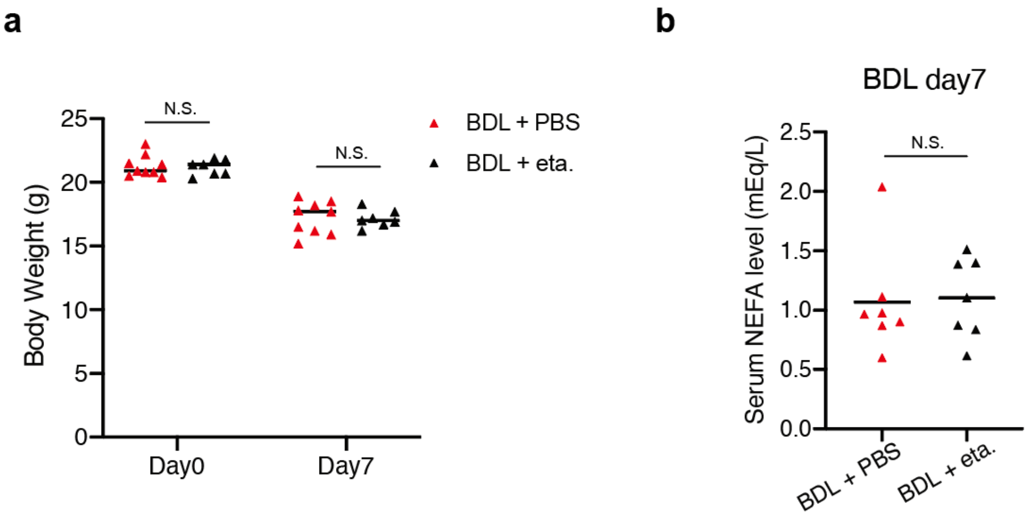

Supplementary Fig. 7

Supplementary Fig. 7: Body weight and serum NEFA levels of BDL mice with or without etanercept treatment

a Body weight of the BDL mice treated with PBS or etanercept was measured at day 0 and 7 post-operation. **b** Serum levels of non-esterified fatty acids (NEFA) of the BDL mice treated with PBS or etanercept were measured at day 7 post-operation. $n = 9$ (BDL + PBS) and 7 (BDL + eta) mice (**a**). $n = 7$ mice for each group (**b**).
